# Fine Mapping and Candidate Gene Identification of *ORUFILM03g000096* Gene in Weedy Rice LM8: Insights into Grain Length Regulation

**DOI:** 10.1186/s12284-025-00858-5

**Published:** 2025-11-05

**Authors:** Fei Li, Zhenyun Han, Leina Zhou, Weiya Fan, Danjing Lou, Jinyue Ge, Yanyan Wang, Ziran Liu, Wenlong Guo, Weihua Qiao, Yunlian Cheng, Lifang Zhang, Danting Li, Baoxuan Nong, Baoqing Dun, Xiaoming Zheng, Qingwen Yang

**Affiliations:** 1 State Key Laboratory of Crop Gene Resources and Breeding, Institute of Crop Sciences, Chinese Academy of Agricultural Sciences, Beijing, 100081 China; 2 Zhongyuan Research Center, Chinese Academy of Agricultural Sciences, Xinxiang, 453500 China; 3 Institute of Botany, The Chinese Academy of Sciences, Beijing, 100093 China; 4 College of Life Science, Shenyang Normal University, Shengyang, 110866 China; 5 National Nanfan Research Institute (Sanya), Chinese Academy of Agricultural Sciences, Sanya, 572024 China; 6 Guangxi Key Laboratory of Rice Genetics and Breeding, Rice Research Institute, Guangxi Academy of Agricultural Sciences, Nanning, 530007 China

**Keywords:** Grain length, Extremely small grains, Quantitative trait locus, Weedy rice, LM8

## Abstract

Although the yield and resistance of cultivated rice have been significantly enhanced since domestication, its genetic basis remains narrow, with many beneficial genes having been lost. Weedy rice is an important source of genetic material for improving rice and provides valuable germplasm resources for identifying excellent genes. In our study, we constructed an F_2_ genetic population by crossing the weedy rice variety LM8, which has extremely small grains, with cultivated rice. High-density genetic maps were constructed using whole-genome sequencing. Five grain shape traits were observed in the F_2_ population, including grain length, grain width, grain thickness, length width ratio and thousand grain weight. The phenotype data were found to be consistent with a normal distribution. Through linkage analysis of the phenotype and genetic maps, a total of 14 QTL loci were identified, one of which was associated with grain length. Grain length values ranged from 5.32 to 11.14 mm. The associated locus was located at 18.81–18.87 Mb on chromosome 3, and accounted for 19.88% of the total phenotypic variation. The BC_1_F_2_ population was constructed using Bulked Segregant Analysis (BSA) sequencing to locate the grain length gene. The *ORUFILM03g000096* gene, which is homologous to the *Os03g0427300* gene of Nipponbare, was identified as the grain length gene. According to the annotation of the known genes, the *ORUFILM03g000096* gene belongs to the Glutelin (GLU) gene family, which is generally associated with rice quality. A systematic investigation of the expression profile of *ORUFILM03g000096* gene using the Rice Expression Profile (RiceXPro) database showed high expression in young panicles. Evolutionary tree analysis revealed a closer evolutionary relationship with the Japanese gene sequence. Hplotype analysis of the gene in the hybrid offspring indicated that a non-synonymous mutation (G-A), located 861 bp downstream of the ATG site in the third exon of the candidate gene, affected the grain length. It is hypothesized that this gene plays a crucial role in grain length growth and enhances rice quality.

**Supplementary Information:**

The online version contains supplementary material available at 10.1186/s12284-025-00858-5.

## Introduction

Grain size characteristics are a crucial determinant of both rice yield and harvest index, and they are regulated by multiple genes. The genetic mechanisms underlying these traits are highly complex (Jiang et al. 2024; Song et al. 2024). To improve rice yield, it is essential to accurately identify and locate the QTL loci associated with grain shape, as well as to discover genes that positively influence yield, which has become the main focus of many researchers (Lu et al. 2023; Du et al. 2025). Quantitative trait loci (QTL) mapping technology has significantly accelerated the identification of genes related to grain shape, and multiple related QTLs have been successfully mapped (Shomura et al. 2008; He et al. 2023; Luo et al. 2025). However, despite substantial achievements in QTL mapping using the linkage map constructed by traditional SSR/RFLP markers, most quantitative traits remain highly susceptible to environmental influences (Takai et al. 2025; Ma et al. 2025). The long mapping cycle and large intervals limit the detection of genetic variation, fine gene mapping and evolutionary analysis. Currently, only a few QTL mapping results can be replicated, and the mapping stability remains a significant challenge. The research into the functions of these loci and the molecular mechanisms involved is very superficial and requires further investigation.

As high-throughput technologies continue to emerge, an increasing number of studies have focused on constructing high-density genetic maps based on Single Nucleotide Polymorphisms (SNPs) (Yan et al. 2020; Asante et al. 2025). Combining this approach with traditional QTL mapping methods can rapidly and effectively detect QTL loci, thereby improving the accuracy of mapping results and further advancing the research process of QTL mapping and the identification of excellent genes in rice seed resources (Lu et al. 2023; Yang et al. 2023). Xie et al. (2010) constructed a high-quality, high-density genetic map using whole-genome resequencing using the Recombinant Inbred Lines (RIL) populations of Zhenshan 97 and Minghui 63. Combined with phenotypic data, they located a *GW5* gene, which controls grain width, within a 200 kb interval. Huang et al. (2013) identified over 400 QTL loci related to rice grain shape, of which 167 were associated with the regulation of thousand grain weight, and 103 and 95 loci were related to the regulation of grain length and width, respectively. Xia et al. (2017) used a backcross population derived from *indica* rice (Jin23B) and *japonica* rice (QingGuAi) to detect ten QTL loci associated with grain shape, including *qGW1*, *qGS3*, and *qGS7*. Zhang et al. (2015a, b) used a population of recombinant inbred lines derived from TD70 and Kasalath to detect 19 QTL loci related to grain shape during QTL mapping. Si et al. (2016) used genome-wide association analysis (GWAS) to identify the *GLW7*, which is related to rice grain shape, for the first time. This locus regulates rice grain shape and yield by encoding the *Os07g0505200* gene, a transcription factor known as *OsSPL13*. Li et al. (2024) found significant natural variations in the *OsCLV2c*, *OsCLV2d*, and *OsCRN1* loci in a genome-wide association study of grain shape in rice, which negatively regulate grain length–width ratio.

The shape of rice grains is a complex trait, including traits such as grain length (GL), grain width (GW), grain thickness (GT), length width ratio (LWR), and thousand grain weight (TGW) (Li et al. 2019; Lu et al. 2023). Studies have identified several key genes that primarily regulate grain length, including *GS2*/*GL2* (Hu et al. 2015; Che et al. 2015) on chromosome 2, *OsLG3* (Yu et al. 2017), *GS3* (Mao et al. 2018), *qGL3*/*GL3.1*/*qGL3-1* (Qi et al. 2012; Gao et al. 2019), *qLGY3*/*OsLG3b*/*GW3p6* (Liu et al. 2018) and *qTGW3*/*TGW3*/*GL3.3* (Hu et al. 2018; Ying et al. 2018; Xia et al. 2018) on chromosome 3, *GL4* (Wu et al. 2017) on chromosome 4, *GL6* (Wang et al. 2019) and *TGW6* (Ishimaru et al. 2013) on chromosome 6, *GLW7* (Si et al. 2016) and *GL7* (Wang et al. 2015a) on chromosome 7 and *GS9* (Zhao et al. 2018) on chromosome 9. These genes are major QTL that regulate grain length and thereby affect yield (Kang et al. 2020; Li et al. 2020). Additionally, genes such as *TGW2* and *GW2* (Song et al. 2007) on chromosome 2, the *GS5* (Li et al. 2011) and *qSW5*/*GW5*/*GSE5* (Liu et al. 2017) on chromosome 5, and the *GW8* (Wang et al. 2012a, b) gene on chromosome 8 primarily regulate grain width and affect grain yield. Other genes, including *GL2*/*GS2* (Hu et al. 2015; Che et al. 2015) on chromosome 2, the *GW6a* (Song et al. 2015) on chromosome 6, and the *GL7*/*GW7* (Wang et al. 2015b) on chromosome 7 control both grain length and grain width and jointly affect yield (Li et al. 2020). Furthermore, it has been found that the *WTG1*/*OsOTUB1* (Huang et al. 2017a, b) on chromosome 8, which has deubiquitinase activity, can regulate grain thickness by controlling the extensibility of glume cells.

Currently, a significant number of studies are focusing on the genes that regulate grain shape in cultivated rice, but relatively few have explored genes in weedy rice. Consequently, establishing a weedy rice mapping population and identifying beneficial genes in weedy rice could be an effective approach to overcome the current breeding bottleneck (Sun et al. 2019; Wu et al. 2022). In this study, we constructed a genetic linkage map of the LM8 weedy rice variety using high-throughput sequencing. This map was applied to a QTL mapping study of five important agronomic traits: GL, GW, GT, LWR and TGW. This map facilitated the identification of genes associated with desirable traits. Concurrently, the BC_1_F_2_ genetic population was created using Shen08S and LM8. The SNP index difference between the large-grain-length (GLL) and the small-grain-length (GLX) pools was calculated based on the allele differences to locate the locus associated with the trait in the genome. This difference was then used to determine the relationship between the positioning interval and grain length formation, as well as to complete the functional annotation of genes within the candidate interval. This study identifies genes related to grain length formation, providing a theoretical basis for the identification of excellent genes involved in grain shape formation.

## Materials and Methods

### Experiment Material

Weedy rice (LM8) was selected from the germplasm collection in the National Gene Bank and obtained through eight consecutive years of self-crossing (Yang et al. 2022). LM8 exhibits a long growth period, a compact plant type, extremely small grains, and good cold resistance (Li et al. 2021; Han et al. 2022). It was cultivated at the Chinese Academy of Agricultural Sciences in 2018. Cultivated rice (Shen08S), provided by the Anhui Academy of Agricultural Sciences, features short plants, a compact plant type, strong tillering ability, and favorable leaf morphology (Hu 2016). The F_2_ generation genetic population (1229 plants), derived from a cross between LM8 and Shen08S, was planted in Nanning, Guangxi Autonomous Region, in the autumn of 2018. Fresh leaves from these plants were harvested and stored at − 80 °C. The BC_1_F_2_ genetic population (1529 plants), constructed by backcrossing the F_2_ population, was planted in Lingshui, Hainan Province, in the winter of 2020. Fresh leaves were collected before heading and stored for subsequent study.

### Investigation of Agronomic Traits

In our experiment, we utilized the Wanshen seed copying instrument to measure the GL, GW, GT, LWR, and TGW for each individual plant in both the F_2_ and BC_1_F_2_ genetic populations (Ma et al. 2016). Subsequently, we conducted correlation analysis of the phenotypes data using R software v3.6.2 (Wang et al. 2006; Langfelder and Horvath 2012). Statistical significance of differences was assessed by one-way analysis of variance (ANOVA) followed by Tukey’s HSD post-hoc test (with adjustment for multiple comparisons), with the significance level set at *p* < 0.05. Correlation analyses were performed using Pearson’s correlation coefficient, and p-values were adjusted using the Benjamini-Hochberg false discovery rate (FDR) procedure.

### Construction of Genetic Map and QTL Mapping Analysis

To improve the accuracy and cost-efficiency of positioning, we randomly selected 199 F_2_ population offspring and their parents (LM8 and Shen08S) with which to construct this genetic map. Extract the genomic DNA from the fresh leaves of the plants using the CTAB method. The purity of the DNA was assessed using a NanoDrop™ One UV-Vis spectrophotometer with an OD260/280 ratio ranging from 1.8 to 2.0 and an OD260/230 ratio ranging from 2.0 to 2.2. The DNA concentration was subsequently measured using a Qubit^®^ 3.0 Fluorometer quantitative instrument (Miao et al. 2024). After quality inspection, qualified DNA samples (350 bp in length) were prepared for library construction (Chen et al. 2018; Luo et al. 2020). Subsequently, the paired-end sequencing method was used to complete the 20x sequencing of the LM8 and Shen08S and the 10x sequencing of the 199 F_2_ offspring using the Illumina sequencing platform (HiSeq PE150) and whole-genome sequencing (Luo et al. 2020).

The BWA software (mem -t -k 32 -M -R) (Xu et al. 2023) was used to compare and analyze the sequencing data for each sample against the reference genome. The SAMtools software was then used to convert the file format, sort the results, and remove duplicates (Li et al. 2009). The GATK software was then used to develop SNP markers for SNP identification and genotyping (Mckenna et al. 2010), and to filter these SNP markers by removing aberrant bases, genotypes and incomplete coverage, as well as by separating distorted markers (Yang et al. 2018). The Joinmap 4.1 software was used to order the marker within each linkage group, and the Perl SVG module was used to visualize the linkage map (Zhang et al. 2015a, b). The quality of the genetic map was assessed using heatmap analysis of the linkage relationships between adjacent markers and monosomy origin analysis (Wang et al. 2020).

### Construction of BC_1_F_2_ Population Library

Seventeen samples were selected from both grain-length-long (GLL) and grain-length-small (GLX) based on the grain length to establish a mixed pool. DNA was extracted from fresh leaves, and the purity of the DNA was detected using a Nanodrop spectrophotometer. The integrity and concentration of the DNA were then detected using a Qubit fluorometer and agarose gel electrophoresis, following the same standards as previously described. After testing the 34 offspring and two parental samples, the genomic DNA of the 36 sequencing samples was processed using ultrasound, during which splicing and repair were completed simultaneously. Finally, agarose gel electrophoresis was used to select the ligated products, followed by PCR enrichment to construct the DNA library.

Prior to sequencing, the quality of the DNA library was assessed using an Agilent 2100 bioanalyzer. The library concentration (> 4nM) was then quantified using the Q-PCR method to complete the quality inspection. Libraries that passed the quality control were pooled. The whole genome shotgun method was then employed to sequence the DNA insert fragments using the HiSeq 4000 (PE150) sequencer. The original sequencing data were processed by removing read pairs containing adapters, sequences with high N content (exceeding 10% of the sequence length), contamination and low-quality reads (Q ≤ 5). The resulting with high quality clean reads were then screened for subsequent analysis.

### Data Comparison Analysis and SNP Frequency Difference Analysis

Using the reference genome sequence of LM8 and clean sequencing data, the valid sequencing data were compared and analyzed using the BWA software (mem -t 4 -k 32 -M) (Xu et al. 2023). Then the alignment results in BAM format were then sorted and deduplicated using the SAMtools software (sort, rmdup), and the alignment rate, coverage depth and genome coverage rate of the data sample were calculated (Li et al. 2009). The GATK3.7 software (Unified Genotyper model) was then used to detect SNP variant sites. The Variant Filtration software was used to filter and screen the SNPs (Mckenna et al. 2010). Finally, the ANNOVA software was employed to perform functional annotation of the filtered gene variants, based on the reference genome annotation information of the LM8.

Based on the genotyping results, sites with resequencing errors and alignment errors (SNP index < 0.3 and SNP depth < 7 in offspring, or missing SNP index sites in one offspring) were filtered out, as well as the homozygous SNP sites between parents. The SNP index of the homozygous sites in the LM8 and Shen08S parents was then calculated in the GLL and GLX offspring pools. The difference in SNP index between the two offspring pools was then calculated (ΔSNP_index = SNP_index (extreme trait B)–SNP_index (extreme trait A) (Takagi et al. 2013). Finally, a 99% confidence level was selected as the screening threshold (*n* = 1000) to determine the candidate interval (Takagi et al. 2013). SNP sites with a significantly different SNP_index in the offspring were selected (one SNP_index ≥ 0.95 and the other SNP_index ≤ 0.05). For the selected candidate polymorphism marker sites, genes that causing non-synonymous mutations and alternative splicing sites were selected as candidate genes, in conjunction with the ANNOVAR annotation file. The assembled LM8 genome was used as the reference genome to annotate these candidate genes for subsequent functional studies.

### Screening of Candidate Genes for QTL Mapping

In the genetic mapping of the F_2_ population, a permutation test (*n* = 1000) was performed using MapQTL 6.0 to determine the LOD value threshold for the following five phenotypic traits: GL, GW, GT, LWR, and TGW (Ooijen et al. 2009). QTL analysis of the trait phenotype was performed using the Composite Interval Mapping (CIM) mapping method in the WinQTL software. These thresholds were used to identify the corresponding QTL segment, their contribution rate and additive effect for the phenotype (Ooijen et al. 2009; Wang et al. 2012a, b). The 99% confidence intervals of the QTLs in the of F_2_ and BC_1_F_2_ populations were identified as candidate intervals. Genes with non-synonymous coding mutations, premature termination mutations, or prolonged termination mutations were considered as functional.

## Results and Analysis

### Comparison of Agronomic Traits Between LM8, Shen08S and the F_2_ Population

**Fig. 1:**
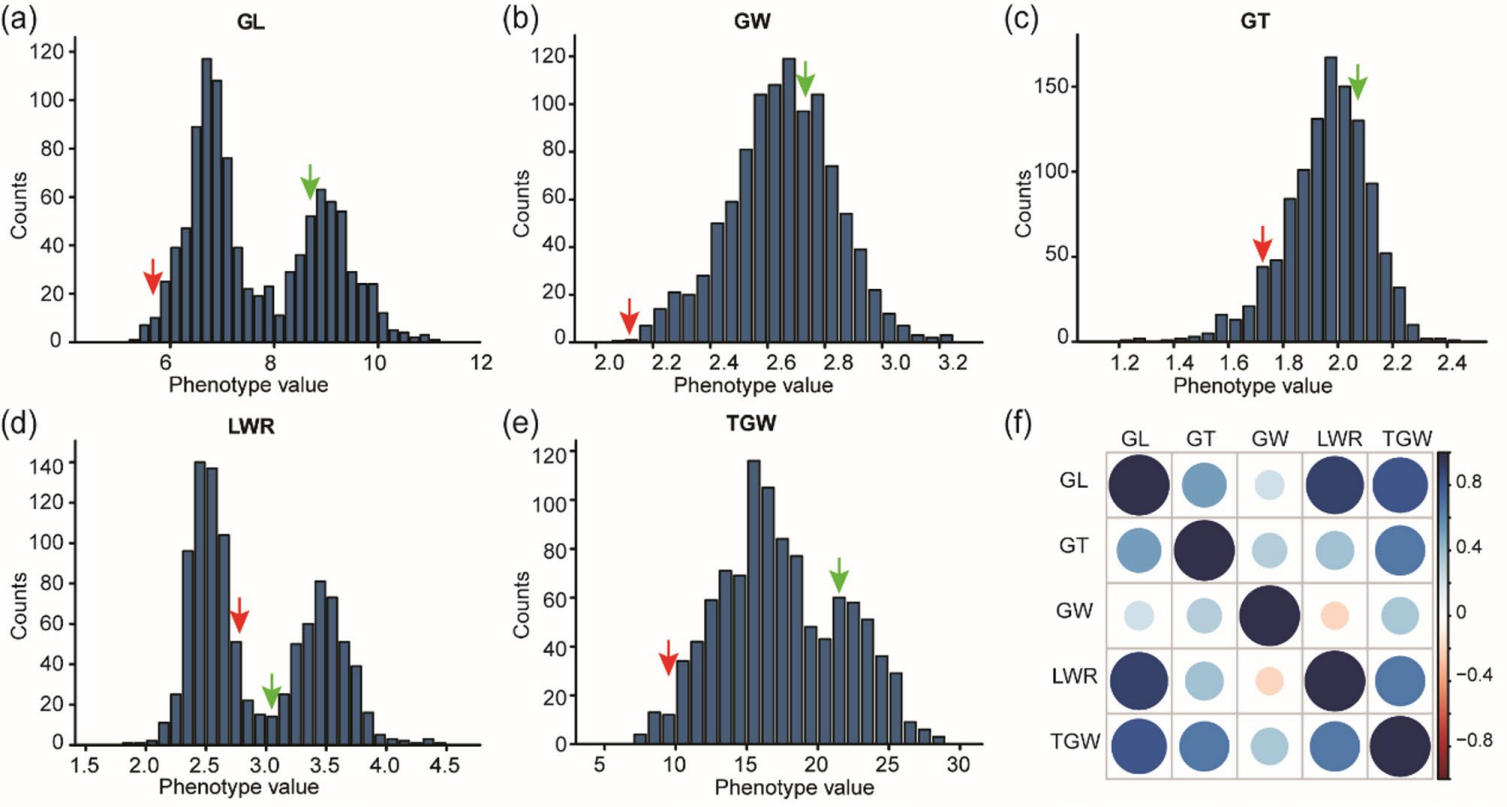
Phenotype distribution and correlation map of the five agronomic traits in the F_2_ population. GL: grain length; GW: grain width; GT: grain thickness; LWR: length to width ratio; TGW: thousand grain weight. The red arrow is parent LM8; Green arrow is parent Shen08S

The agronomic performance of the two parent plants and the F_2_ population was evaluated by measuring the agronomic traits. Analysis of the phenotypic data revealed that the weedy rice varieties LM8 had a GL of only 5.81 mm and a TGW of 10.32 g, both of which were significantly smaller than those of the cultivated rice variety Shen08S, which had a TGW of 22.13 g. In the F_2_ population, five important agronomic traits were measured: GL ranged from 5.5 to 11 mm, GW from 2.1 to 3.2 mm, GT from 1.2 to 2.4 mm, LWR from 2 to 4.5, and TGW from 7 to 28 g. These traits exhibited an approximate normal distribution (Fig. 1), which is suitable for QTL mapping.

Correlation analysis of five grain traits (GL, GW, GT, LWR and TGW) in a population of 1229 F_2_ plants revealed a negative correlation between GW and LWR (− 0.198), while the other traits showed positive correlations (Fig. 1f; Table 1). Further analysis of the five traits controlling grain shape revealed extremely high correlations between GL and LWR (0.91), and between GL and TGW (0.83) (Table 1). Our study is consistent with previous research conclusions that genes such as *GS3*, *GL7* and other grain length genes, are positively correlated with thousand grain weight (Fan et al. 2006). Therefore, exploiting the extremely small grain shape of LM8 to identify genes that control grain shape is crucial for enriching rice germplasm resources.

**Table 1:** Correlation statistics of five grain shape traits of weedy rice varieties LM8

	GL	GT	GW	LWR	TGW
GL	1	0.528	0.224	0.908	0.829
GT	0.528	1	0.322	0.395	0.673
GW	0.224	0.322	1	-0.198	0.368
LWR	0.908	0.395	-0.198	1	0.674
TGW	0.829	0.673	0.368	0.674	1

GL: grain length; GW: grain width; GT: grain thickness; LWR: length to width ratio; TGW: thousand grain weight

### QTL Mapping for Grain Shape Based Genetic Map

**Fig. 2:**
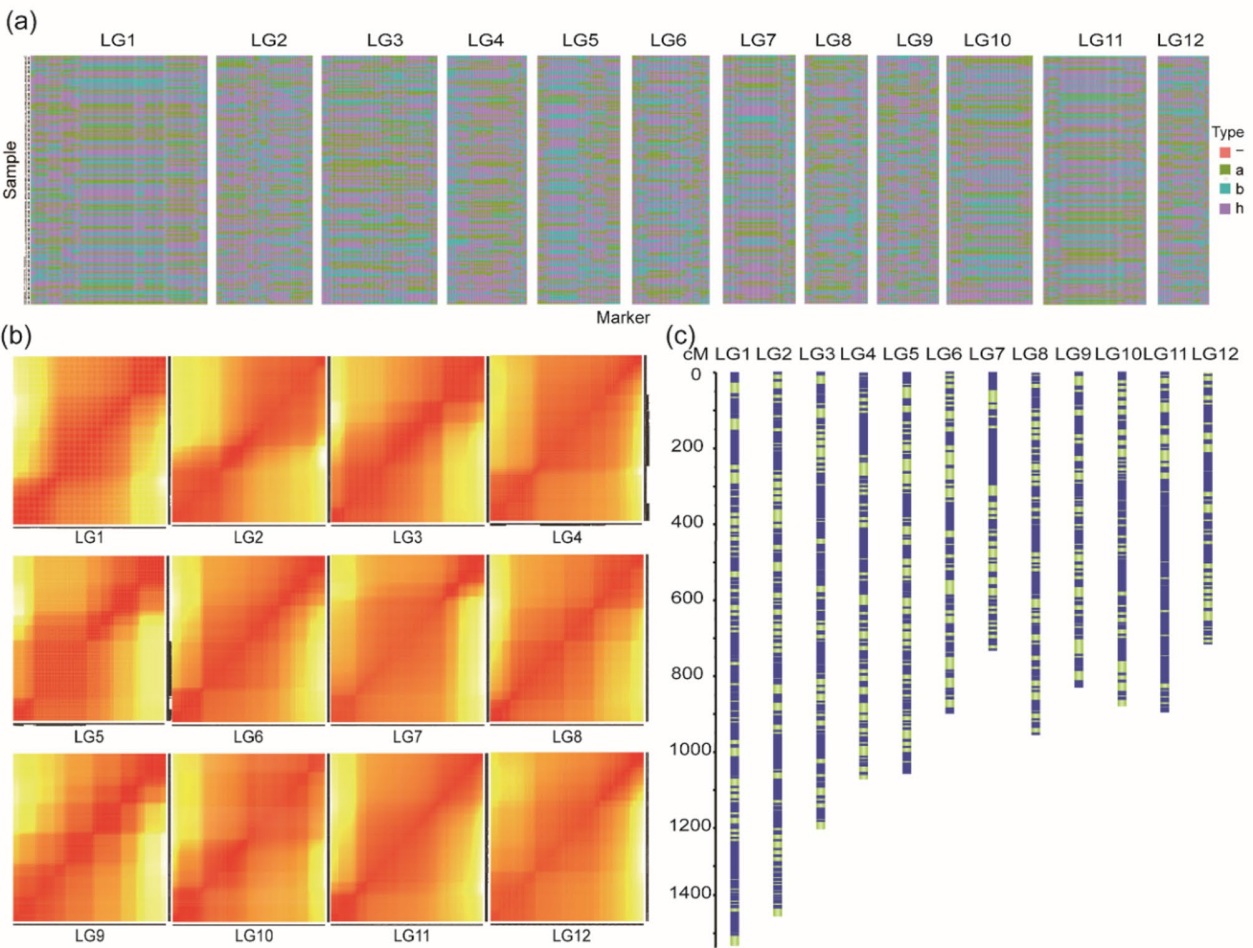
Genetic map constructed based on the F_2_ population. **a** Distribution map using monosomy origin. **b** Heatmap analysis between genetic markers. **c** Genetic map of the F_2_ population. LG1-LG12 represent the 12 chromosomes

Linkage analysis between a genetic map and phenotypic data can rapidly identify new genes and beneficial alleles within germplasm resources, thereby enriching them (Li et al. 2021; Cai et al. 2025). In our experiment, genetic markers were evenly distributed across 12 chromosomes (Fig. 2c). The quality of the constructed genetic map was evaluated using a heatmap analysis of the linkage relationships between the monosomy origin analysis (Fig. 2a) and the adjacent markers (Fig. 2b). The results showed that the linkage strength between adjacent markers was higher than that between distant markers, and that the origin of most segments within each individual remained consistent. These results suggest that the map markers are relatively uniform and of high quality, making the genetic map suitable for further research.

**Fig. 3:**
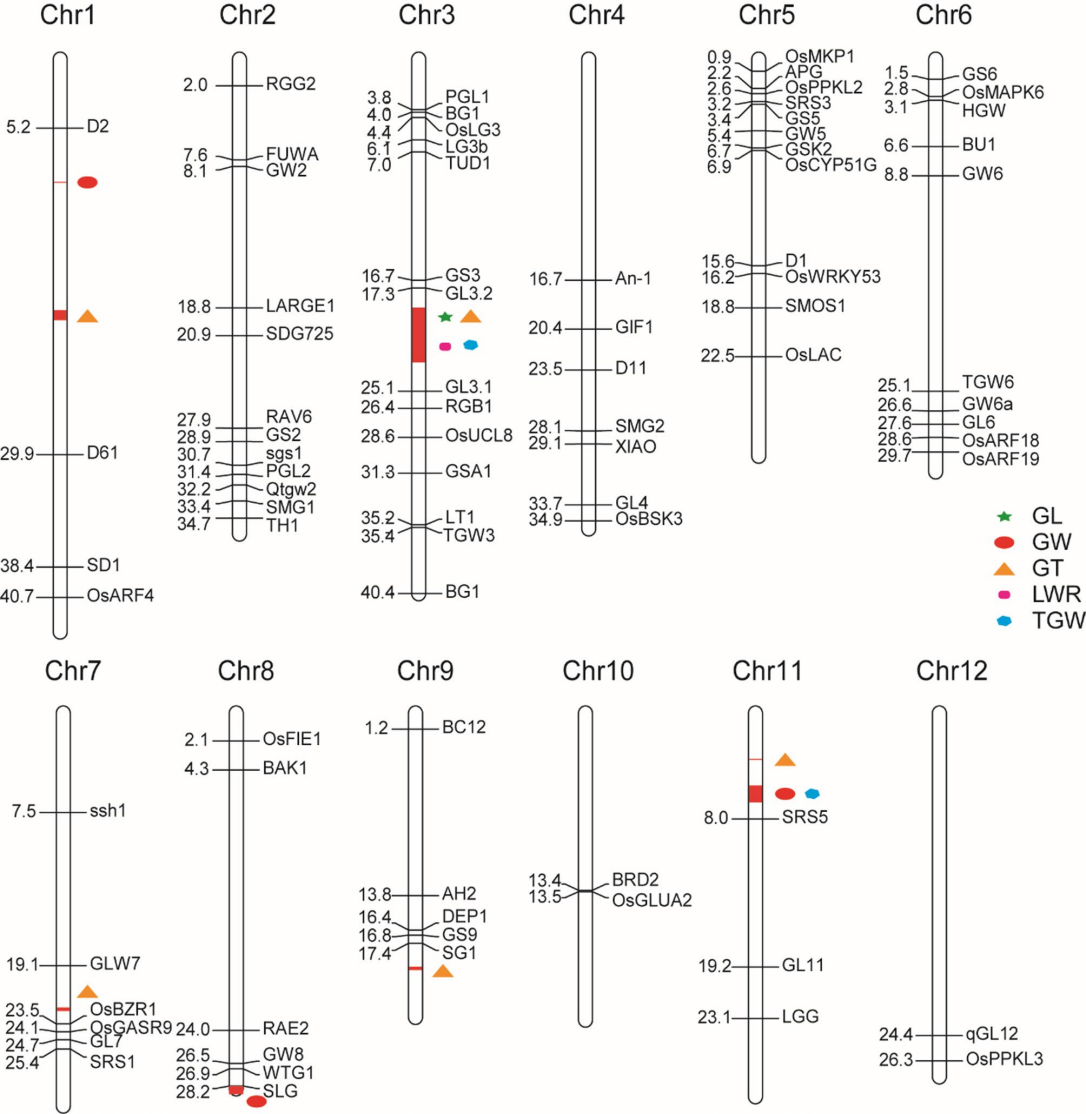
QTL mapping of grain shape in F_2_ population and distribution of cloned grain shape genes on 12 chromosomes. Chr1-chr12 represent the 12 chromosomes. The colored symbols on chromosomes represent different traits: Green pentagram: GL; Light red ellipse: GW; Orange triangle: GT; Purple rounded rectangle: LWR; Blue hexagon: TWG. GL: grain length; GW: grain width; GT: grain thickness; LWR: length to width ratio; TGW: thousand grain weight

Currently, 83 genes related to grain shape have been cloned (Fig. 3). In our experiment, we conducted linkage analysis between the phenotypic data and a genetic map for five grain shape traits in the F_2_ population. This analysis identified 14 QTL loci associated with these five grain traits (Fig. 3; Table 2). Specifically, one, three, six, one and three loci were identified as regulating GL, GW, GT, LWR and TGW, respectively. Further analysis revealed that five of these loci had a contribution rate of over 17% (major QTL loci; Jin et al. 2025a, b), which were located on chromosomes 3 (782.9-786.6 cM, 787.6-788.2 cM and 788.3-789.4 cM) and 11 (34.4-37.5 cM and 244.6-253 cM; Fig. 3; Table 2). The remaining nine loci were minor-effect QTLs, which, together with the major loci, collectively influence grain shape. These results provide the foundation for the future studies aimed at enriching rice germplasm resources.

According to the QTL mapping results and genome annotation information, we identified 290 genes related to grain shape traits that are located within the fourteen QTL loci. The *ORUFILM03g004244* gene, which is associated with thousand grain weight, belongs to the *hak21* gene family of high-affinity potassium ion transporters, as dose the *Os03g0576200* gene in Nipponbare. The *ORUFILM03g000096* gene, which affects grain length, has a homologous gene (*Os03g0427300*) in Nipponbare that belongs to the *glua* gene family, which is involved in gluten formation. The *ORUFILM09g001504* gene, which responds to grain thickness and has a homologous gene (*Os09g0492700*) in Nipponbare that belongs to the *hmg2* gene family, which is associated with high-mobility proteins. Furthermore, given the unique characteristics of the extremely small grain shape of weedy rice LM8, we examined the correlation coefficients of the five phenotypic traits and the contribution rates of the QTL mapping sites to phenotypic variation. We found that the grain length of LM8 was only 5.81 mm, contributing a rate of 19.87% to phenotypic variation. This trait was positively correlated with thousand grain weight (*r* = 0.83), suggesting that grain length is a key point to consider in further studies.

**Table 2:** QTL mapping of grain shape in F_2_ population

Trait	Chromosome	Geneetic interval (cM)	Physical interval (Mb)	Position (cM)	LOD	Additive		PVE (%)
GL	LG03	788.3-789.4	18.81-18.87	788.51	12.664495	0.4535		19.8757
GW	LG01	413.4-426.2	9.33-9.34	422.11	5.954397	0.0758		6.204
GW	LG08	184.3-197.7	28.76-29.22	184.61	9.680782	0.106		12.6482
GW	LG11	243.3-269.6	5.52-6.69	252.01	6.703583	−0.0924		12.7571
GT	LG01	655-661.3	19.04-19.1	657.81	4.102063	0.0409		10.1391
GT	LG01	1060.4-1078.5	19.65-19.67	1064.41	4.184582	−0.1041		0.0001
GT	LG03	785.1-789.4	18.81-18.93	788.51	4.197611	0.0284		8.3976
GT	LG07	413.8-427.3	22.32-22.52	419.91	4.612378	0.0577		6.899
GT	LG09	762.9-780	19.17-19.36	764.91	5.224756	− 0.0419		0.9647
GT	LG11	34.4-37.5	3.48-3.48	36.11	6.123779	− 0.0563		17.3557
LWR	LG03	764.3-766.2	19.34-19.35	765.51	10.015201	0.2307		0
TGW	LG03	782.9-786.6	22.87-22.91	785.61	22.749186	2.7575		34.7431
TGW	LG03	787.6-788.2	18.85-18.87	787.91	23.897937	2.7714		36.487
TGW	LG11	244.6-253	5.52-5.53	251.01	11.589577	− 2.5234		18.845

GL: grain length; GW: grain width; GT: grain thickness; LWR: length to width ratio; TGW: thousand grain weight. LOD: the LOD value of this QTL. PVE: phenotypic variance explained

### Mapping of Candidate Gene Based on BSA-seq in BC_1_F_2_ Population

Using the Bulked Segregant Analysis (BSA) sequencing research method, we first sequenced four samples (LM8, Shen08S, GLL and GLX) to analysis the quality distribution of the sequencing data. The results showed that the Q20 quality values for all four samples were higher than 97%, the error rate for each sample was less than 0.4%, and the GC content ranged from 41.58% to 43.58%. There was no separation phenomenon of AT and GC, and the proportions of A and T, as well as C and G were relatively consistent. In each sequencing sample of LM8, Shen08S, GLX and GLL, the clean data available for analysis accounted for more than 98%, and the coverage of the reference genome was greater than 97%. The bases coverage exhibited high integrity and depth, with at least 4X coverage accounting for 92.96%, 84.79%, 91.13%, and 91.67%, respectively.

Variation detection in the sequencing data revealed a total of 2,525,828 SNP loci. A detailed statistical analysis of the positional information of these variant sites revealed the following number of SNPs: intergenic, 1,435,768; upstream, 210,815; downstream, 175,636; upstream of one gene and downstream of another gene (upstream/downstream), 21,881; intron, 517,632; splicing, 535. Among these SNPs, 1,783,921 were transitions, and 741,907 were transversions. Meanwhile, 16,351 mutations occurred in the exon region: 1,678 gained stop codons, 411 lost stop codons, 90,447 non-synonymous, and 70,985 synonymous mutations, respectively. Additionally, 40 sites had other unknown functional variants. Based on the genotyping results, a total of 1,739,119 polymorphic marker sites were initially screened from the SNP sites of the parental homozygotes. After filtering out incorrect bases and low-quality bases, 65,105 high-reliability polymorphic sites were obtained for further analysis.

**Fig. 4:**
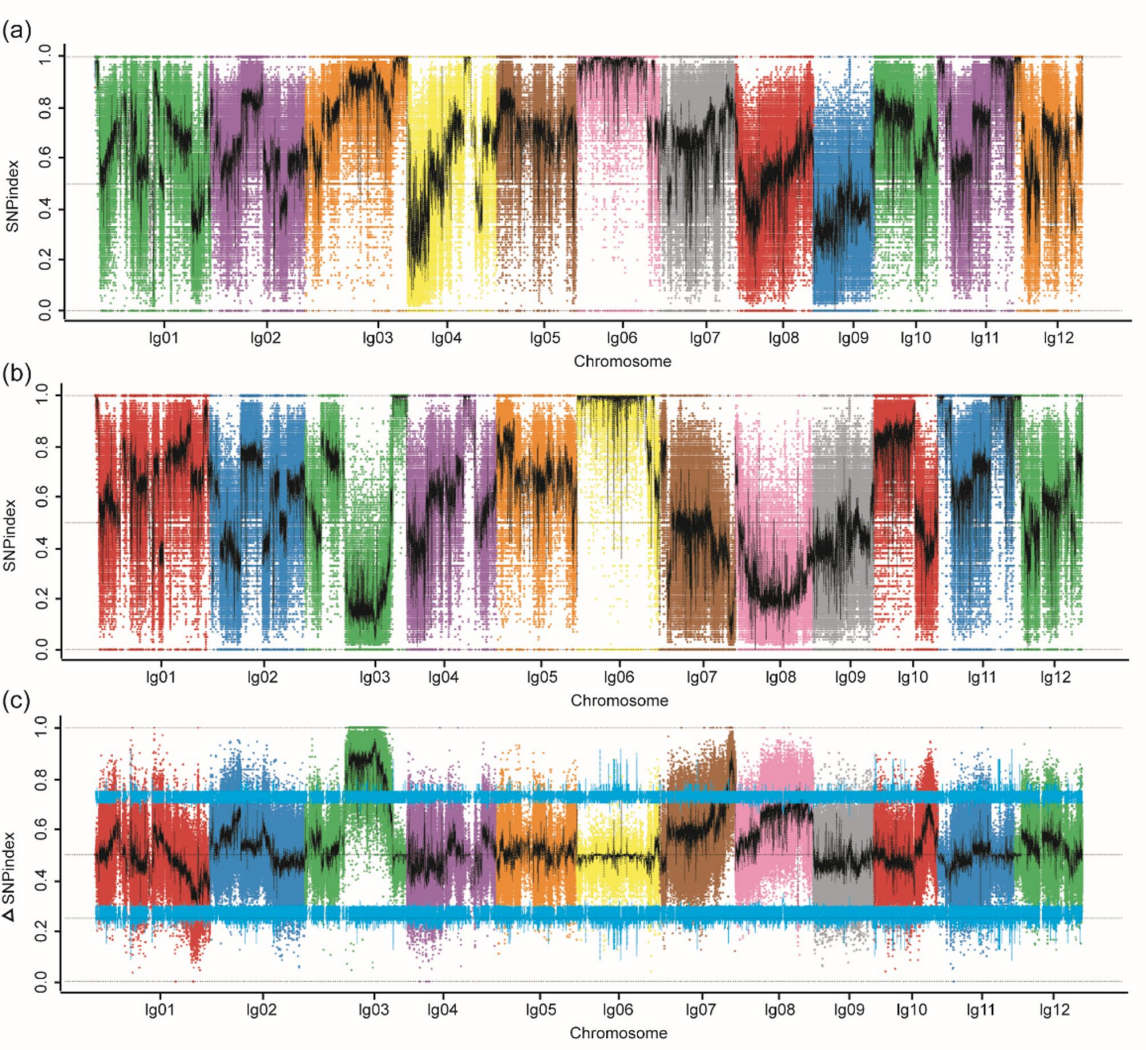
Distribution of SNP index on chromosome. **a** SNP-index of GLL. **b** SNP-index of GLX. **c** ΔSNP-index. lg1-lg12 represent the 12 chromosomes. SNP-index: the proportion of reads containing a mutant parent at a given locus relative to the total number of reads. ΔSNP-index represents offspring frequency difference

The SNP-index indicates the proportion of reads containing a mutant parent at a given locus relative to the total number of reads. The strength of the correlation between the SNP marker and the trait indicates the value of the ΔSNP-index. Intervals exceeding the threshold are typically identified as candidate intervals associated with the trait. The distribution of the GLL and GLX pools SNP-index is presented in Fig. 4a and b. The experiments findings demonstrated that, at a 99% confidence level, 77,223 variation sites were detected, and three localization intervals were obtained. The distribution of these variation sites across the genome is illustrated in Fig. 4c, with the relevant chromosomal locations specified as chromosomal 3 (15,566,462-32,481,696), chromosomal 7 (25,720,805-28,906,106) and chromosomal 8 (20,072,235-27,375,244). The mapping interval on chromosome 3 encompasses the QTL mapping interval of the F_2_ population, thereby further substantiating the precision of the mapping results. Subsequent analysis of the candidate genes within the candidate intervals revealed 2734 genes with non-synonymous mutations, 2094 genes with synonymous mutations, 49 genes with mutations that gained stop codons, and 9 genes with mutations that lost stop codons.

In accordance with the findings of the 99% confidence interval and the SNP-index being greater than or equal to 0.95 (or less than or equal to 0.05), 3,619 SNP variant sites were identified at the intersection of two genes. This set of genes includes 88 genes with synonymous mutations in exons and 73 genes with non-synonymous mutations among the 126 non-synonymous mutated SNP sites. The annotated candidate genes were derived from the Non-Redundant Protein Sequence Database (NR), Swiss-Prot Protein Sequence Database (Swiss-Prot), Gene Ontology (GO), Kyoto Encyclopedia of Genes and Genomes (KEGG) and EuKaryotic Orthologous Groups (KOG) databases, with 72, 42, 53, 11, and 26 genes annotated in each database, respectively.

### Analysis of Candidate *ORUFILM03g000096* Gene of Grain Length

**Fig. 5:**
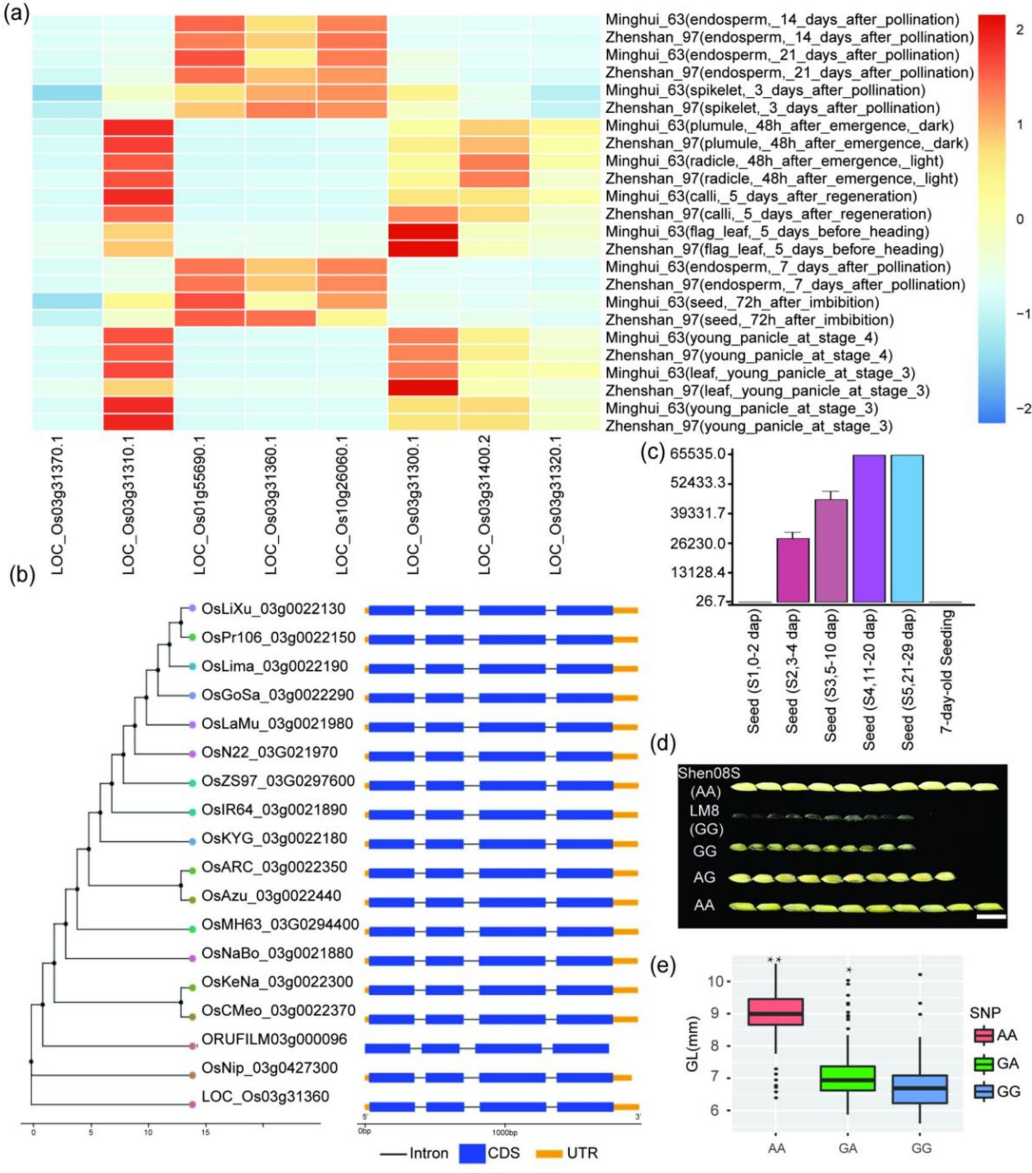
Gene expression, evolutionary tree, and phenotype analysis of *ORUFILM03g000096*. **a** Heat map of annotated genes in the region of candidate gene. Gene expression profile data were obtained from the Collections of Rice Expression profiling (CREP) database. Red indicates high expression, and blue indicates low expression. **b** Gene evolutionary tree analysis of *ORUFILM03g000096* gene in *Oryza sativa*. The analysis of homologous gene evolutionary tree was completed using the Rice Gene Index database. **c** Expression patterns of the candidate gene. Gene expression profile data were obtained from Rice Expression profile (RiceXPro) database. **d** Phenotypes of parents and hybrid offspring. **e** Grain length (GL) distribution in each genotype of *ORUFILM03g000095*. Asterisks indicate the correlation between genotype and phenotype, *P < 0.05. **P < 0.01

The grain length gene has been shown to significantly increase thousand grain weight and yield (Wang et al. 2015a) and is also closely related to rice quality, which can be improved by optimising grain shape. Non-synonymous mutations typically result in changes to gene function. Therefore, we speculate that the candidate genes containing non-synonymous mutations that were selected in this study play a key role in regulating grain development. We combined the results of localising BSA sequencing data and the genetic map localisation results, combined with sequence alignment analysis of coding or regulatory regions, homologous sequence alignment analysis, and gene function prediction analysis. A total of seven candidate genes were identified within the localisation interval on chromosome 3, including *ORUFILM03G000091*,* ORUFILM03g000092*, *ORUFILM03g000093*, *ORUFILM03g000094*, *ORUFILM03g000095*, *ORUFILM03g000096* and *ORUFILM03g000097* (Tabel S1). Based on the localisation results and annotation information, the *ORUFILM03g000096* gene was found to be homologous to the *Os03g0427300* (*LOC4333164*) gene in Nipponbare. This gene belongs to the GLU gene family. It is widely believed that this family of genes is related to gluten formation and affects grain size. To further discriminate between the candidate genes, the expression patterns of all the predicted genes in the region were analyzed during the young panicle and grain development stages using the Collections of Rice Expression Profiling (CREP) database (https://crep.ncpgr.cn/) (Fig. 5a). Evolutionary tree analysis of the *ORUFILM03g000096* gene in the rice homologous gene cluster, conducted using the Rice Gene Index (RGI) database (https://riceome.hzau.edu.cn/), revealed a higher degree of similarity and a closer evolutionary relationship with the Japanese gene sequence (Fig. 5b). A thorough investigation into the expression profiles of the *ORUFILM03g000096* gene was conducted using the Rice Expression Profile (RiceXPro) database (https://ricexpro.dna.affrc.go.jp/). This investigation revealed high levels of expression in young panicles, as illustrated in Fig. 5c.

To further investigate the impact of genetic variation on phenotype, we analyzed the *ORUFILM03g000096* gene sequence from LM8, Shen08S, and their progeny. This analysis revealed three SNP mutation sites in the coding region, but without InDels mutation (Tabel S2). One non-synonymous mutation (G-A) was located 861 bp downstream of the ATG site in the third exon results in an amino acid mutation. Prediction and analysis of protein structural domains revealed that the affected residue is located within a conserved functional domain and is itself highly evolutionarily conserved across monocots. Grain length in the F_2_ individuals of LM8 and Shen08S exhibited a distinct pattern, with AA exhibiting the greatest length, followed by GA and then GG (Fig. 5d). Haplotypes analysis of this gene in the hybrid progeny revealed that different non-synonymous mutations in the CDS region result in different phenotypes within the population. Based on an analysis of representative genotype results combined with population results, offspring with the GG genotype, like LM8, have a similar grain length similar to that of LM8, while offspring with the AA genotype, like Shen08S, have a similar grain length similar to that of Shen08S. However, the grain length of heterozygous offspring with the GA genotype is intermediate between the two parental phenotypes (*P < 0.01*; Fig. 5e). It is speculated that variation in this gene may affect grain shape formation. Furthermore, the *ORUFILM03g000096* gene is hypothesized to play an important role not only in grain length growth but also in improving rice quality.

### Haplotype Analysis of Homologous Gene

**Fig. 6:**
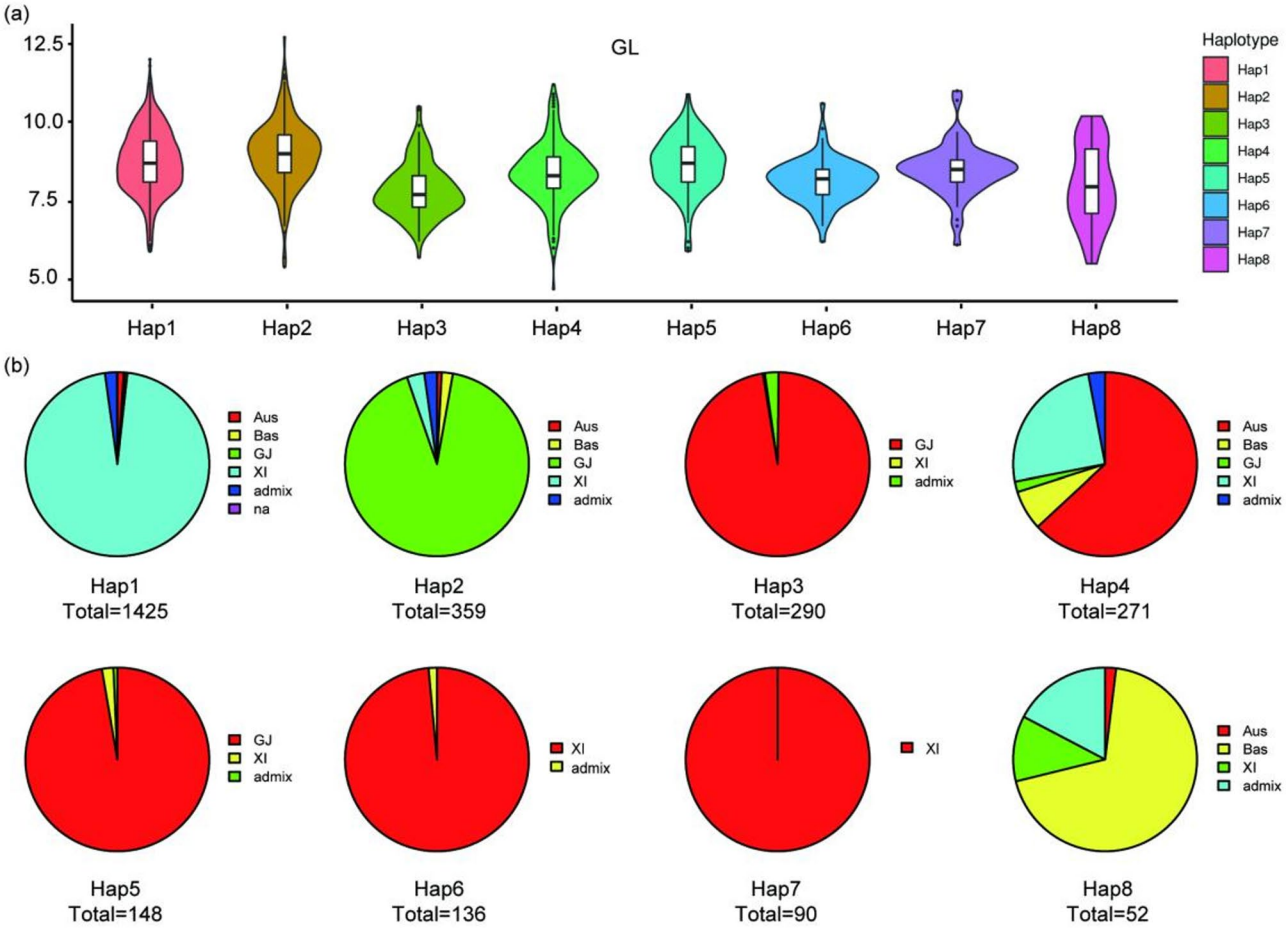
Haplotype analysis for the candidate gene *Os03g0427300*. **a** Violin diagram of GL across different sites in approximately 3000 natural rice accessions. **b** Proportion of eight haplotypes in different rice population. Aus: Aus population; Bas: Basmati population; GJ: geng/*japonica* population; XI: xian/*indica* population; Admix: admixed between any two or more of the XI, GJ, Aus, Bas populations

We performed a haplotype analysis on the genome sequence of the homologous gene *Os03g0427300* in cultivated rice germplasm using resequencing data from the Rice Functional Genome Breeding database (RFGB) (http://www.rmbreeding.cn/Index). Based on the 14 SNPs in the coding region, the gene has eight major haplotypes, ranging from Hap1 to Hap8 (Fig. 6a). Hap1 encompasses the largest number of rice germplasm varieties, totaling 1425, with 1369 XI types, accounting for 96%. Hap3 and Hap5 mostly comprise GJ-type varieties, with 290 and 148 rice germplasm varieties, respectively. Hap4 contains 271 germplasm materials, of which the Aus variety is the most abundant, accounting for 63%. Both Hap6 and Hap7 have the highest proportions of XI types:134 out of 136 germplasm samples in Hap6 are XI materials, and all materials in Hap7 are XI varieties. Hap8 contains a total of 52 materials, with Bas type materials being the most common. Hap2 has the longest grain length at 8.99 mm, while Hap3 has the shortest at 7.83 mm (Fig. 6b). Additionally, materials with Hap2 have longer grain lengths than those with Hap3.

## Discussion

### Hybridization of Weedy Rice and Cultivated Rice To Broaden the Genetic Resources of Cultivated Rice

Excellent rice varieties are crucial for increasing rice yield, improving taste and quality, and enhancing resistance to pests and diseases. However, artificial selection and natural variation during domestication have affected rice, leading to a gradual decrease in genetic diversity, and creating a bottleneck in breeding efforts (Ma et al. 2025). Zhao et al. (1999) examined 10 *indica* and *indica* rice hybrids, 15 *japonica* and *japonica* rice hybrids, and 30 *indica*-*japonica* rice hybrids, finding that the heterosis in inter-subspecies hybrids was significantly higher than in intra-subspecies hybrids. In a study of 1495 rice hybrids and their parents, genotyped using resequencing data, Huang et al. (2015) found that overall heterozygosity of the whole-genome genotypes had little impact on heterosis. Instead, the correlation between the number of heterozygous locus and hybridization performance was relatively weak. However, a stronger correlation was observed between the number of accumulated superior gene alleles at an effective loci and hybridization performance. Previous studies on excellent genes in Asian cultivated rice have primarily focused on the *indica*-*japonica* hybridization, which has a relatively close genetic relationship and thus limited the creation of new germplasm. Chinese and international gene banks provide abundant germplasm resources that are essential for future crop improvement. Currently, approximately 7.4 million germplasm resources are stored in around 1,750 plant germplasm banks worldwide, yet fewer than 2% of these have been utilized as Plant Genetic Resources (PGR) for crop improvement (Janzen et al. 2019). *Oryza rufipogon* is often incompatible with cultivated rice during hybridization, posing significant challenges for breeding. In contrast, weedy rice is widely distributed and closely related to cultivated rice, with highly compatibility and a broad genetic basis. Its natural growth environment avoids artificial selection pressure and contains a large number of beneficial genes. Crossbreeding with cultivated rice can effectively broaden the latter’s genetic basis, introduce new, excellent alleles into the rice germplasm resource pool and greatly enhance genetic diversity.

Rice does not necessarily evolve to have large grains under natural growth conditions. The process of grain shape formation is relatively complicated, with grain length being the most important factor in determining grain shape (Zhan et al. 2022). Different varieties often have grain lengths ranging from approximately 6 to15 mm. Currently, rice grains in China are categorized into four types based on grain length: extremely long grain (> 9.1 mm), long grain (8.1-9.0 mm), medium long grain (7.1-8.0 mm) and short grain (< 7.0 mm) (Cheng et al. 2021). The grain length of LM8 (5.71 mm) is significantly shorter than the 7 mm characteristic of the short-grain rice. Therefore, it is valuable to use this weedy rice to study the genes responsible for grain length formation, as it can broaden research on cultivated rice. In our study, we crossed cultivated rice with weedy rice to obtain an F_2_ population, thereby introducing excellent genes from the weedy rice into the cultivated rice. Among the 14 QTL loci, five loci contribute more than 17% to the phenotypic variation of the plants, and 290 candidate genes were identified. Candidate genes associated with the regulation of grain thickness (*ORUFILM09g001487*), grain width (*ORUFILM08g002572*) and thousand grain weight (*ORUFILM03g004239*) were significantly enriched in twenty pathways related to catalytic activity, proteolysis and transmembrane transporter activity, playing a crucial role in plant growth and development. Acquiring these candidate genes is highly significant for the effective utilization the excellent genes found in weedy rice germplasm and for enriching the narrow genetic basis of cultivated rice. However, due to weedy rice’s characteristics of low yield, poor plant type, shattering and high sensitivity, many excellent traits are difficult to utilize directly, and there are still some deficiencies in practical application. Genetic improvement is needed to enable more effective application in rice breeding research, which remains a top priority for future research (Yang et al. 2006).

### The Significance of BSA Localization Analysis for Excavating Candidate Genes in Rice

BSA mapping is an efficient method of genetic mapping based on the extreme phenotypes in genetically isolated populations (Tang et al. 2024), which is analyzed by constructing extreme mixed pools, and significantly reduces experimental costs and workload while improving the efficiency of linked marker (Li and Xu 2021). It has been successfully applied to populations such as double haploids (DHs) and recombinant inbred lines (RILs), and is widely used for trait gene mapping in different species, demonstrating its broad applicability (Kurlovs et al. 2019). Not only is BSA mapping applicable to qualitative traits controlled by major genes, it can also effectively locate complex quantitative traits (Aoun et al. 2017; Li et al. 2022). The key lies in focusing on the allele frequency differences of target genes in extreme populations rather than their distribution throughout the entire population to achieve more efficient and accurate screening of target loci (Li and Xu 2021). In our experiment, we constructed a mixed pool using two extreme materials with extremely materials with significantly different grain lengths and employed BSA mapping to screen the location interval showing differences on chromosome 3. We identified 73 non-synonymous genes in this region that could be potential candidate genes for regulating grain growth.

The efficiency of BSA in detecting QTLs depends on the heritability of the target traits, the size of the genetically segregating population and the sequencing depth (Guo et al. 2016). The precise localization strategy that combines BSA and high-throughput sequencing has significantly broadened the scope of gene mapping research. However, most BSA-based localization research primarily focuses on preliminary studies, resulting in large candidate intervals. Our BSA localization study found a candidate interval on chromosome 3 that encompasses the genetic map location interval, thereby validating the genetic map location results. However, the candidate location interval (16.9 cM) remains relatively large. Therefore, a larger sequencing population needs to be constructed for BSA population to further refine the positioning results. Meanwhile, SNP-based KASP markers could be designed for the large candidate interval to help to narrow down the positioning interval (Huang et al. 2017a, b). Additionally, screening differentially expressed genes within the candidate interval using combined transcriptomics analysis can further reduce the range of candidate genes (Baek et al. 2017; Wen et al. 2019).

### Candidate Gene Prediction Analysis Facilitates Rapid Excavating of Gene Function Research

Given the abundant rice germplasm resources in China, the rapid identification of candidate genes in these resources is crucial for the accelerated and accurate molecular design of breeding programs. Accurate prediction of candidate gene provides a reliable foundation for subsequent gene function verification. Generally, comparing differences in DNA sequences between the coding and regulatory intervals is a common method of screening candidate genes. By comparing differences in the coding region, Xiong et al. (2018) found that the variation in the *OsLG3* gene sequence led to amino acid changes, resulting in premature translation termination and the failure to form normally expressed proteins, thereby affecting gene function. Li et al. (2011) found that variation in the *GS5* promoter region caused changes in gene expression, which in turn affected the expression of related pathway genes. Meanwhile, some studies have used quantitative expression analysis to detect the relationship between differences in gene expression and target traits to predict candidate genes, and subsequently examine the relationship between genes and proteins (Fujisaki et al. 2004). Lei et al. (2020) screened the differentially expressed candidate gene *qRSL17* for salt tolerance in rice using a combination of transcriptome analysis and QTL mapping information. Additionally, they used gene sequence homology between different species to screen for functional similarity between species via colinear analysis. For species with reference genomes, genome annotation information was employed to screen for candidate genes (Shi et al. 2025).

In our study, we comprehensive analysis of the seven candidate genes related to grain length formation within the target interval of the results of the QTL and BSA mapping found that the *ORUFILM03g000096* was identified as the key gene with the greatest potential for breeding applications. Firstly, this gene encodes gluten, an important storage protein in rice seeds. Although glutelin do not directly control grain length, glutelin and grain length are traits that are genetically and physiologically correlated. Glutelin serves as the primary source of nitrogen and amino acids stored in rice seeds to support seed germination and early seedling growth. It is broken down by proteases into amino acids, providing essential nutrients and energy for seedling development, which may indirectly influence grain length. Furthermore, the glutelin protein genes are often located in close proximity on chromosomes to grain shape genes (Fan et al. 2006). Due to genetic linkage and the dilution effect, long-grain varieties tend to be associated with relatively lower glutelin content, whereas short-round grain varieties may exhibit higher glutelin levels (Jin et al. 2025a, b). Secondly, expression analysis revealed significant differential expression patterns in developing grains and a potential functional non-synonymous SNP mutation in the coding region, suggesting that this mutation may affect protein function and thus the phenotype. Furthermore, haplotype analysis based on the 3000 rice genome revealed a significant association between this gene’s allelic variations and grain length phenotype, providing population genetic evidence that supports its regulation of grain length. Multidimensional evidence indicates that the *ORUFILM03g000096* gene is a key candidate for controlling grain length and quality formation and has significant application value in rice molecular breeding. However, the exact molecular mechanism of candidate genes still requires further analysis. Subsequent research could involve functional validation experiments, such as gene knockout, overexpression and genetic complementarity (Yu et al. 2023), combined with spatiotemporal expression profiling and protein interaction network analysis. This would help to elucidate the biological function and mechanism of action of the gene in a systematic way, providing a theoretical basis and technical support for rice genetic improvement.

## Conclusion

In the study, we constructed a genetic population of F_2_ by crossing the weedy rice variety LM8, which has extremely small grains, with cultivated rice. Five grain shape traits, including GL, GW, GT, LWR and TGW, were observed in the F_2_ population, and a total of 14 QTL loci were identified.

Grain length values from the LM8 weed rice were extremely small, ranged from 5.32 to 11.14 mm. To fine mapping the genes responsible for the excellent grain length, we utilized a localization analysis of the BC_1_F_2_ population and identified the relevant region as being located at 18.81–18.87 Mb on chromosome 3. This region accounted for 19.88% of the total phenotypic variation. The grain length gene, *ORUFILM03g000096* gene, which is homologous to the *Os03g0427300* gene of Nipponbare, was identified as the grain length gene. The *ORUFILM03g000096* gene belongs to the *glua* gene family, which is generally associated with rice quality, and showed high expression in young panicles. A non-synonymous mutation (G-A), located 861 bp downstream of the ATG site in the third exon of the candidate gene, affected the grain length. The non-synonymous SNP marker is highly associated with the long-grain phenotype and provides a valuable genetic resource and a precise molecular tool for marker-assisted selection (MAS). The *ORUFILM03g000096* gene was considered as a novel and promising candidate gene for grain length regulation, which would be highly significant potential for rice molecular breeding.

## Supplementary Information

Additional file 1.

## Data Availability

The original contributions presented in the study were included in the article. For further information, please contact the corresponding authors.

## References

[CR1] Aoun M, Kolmer JA, Rouse MN, Chao S, Acevedo M (2017) Inheritance and bulked Segregant analysis of leaf rust and stem rust resistance in durum wheat genotypes. Phytopathology 107:1496–1506. 10.1094/phyto-12-16-0444-r 28745102 10.1094/PHYTO-12-16-0444-R PMC7779972

[CR2] Asante MD, Ofosu KA, Frimpong F, Alphonso DK, Nartey E, Obeng AE, Bam RK, Gamenyah DD, Ribeiro PF, Manilal W (2025) Effectiveness of KASP-SNP markers in selecting for grain quality traits in rice. Plant Gene 43:100503. 10.1016/j.plgene.2025.100503

[CR3] Baek G, Kim CW, Kim S (2017) Development of a molecular marker tightly linked to the C locus conferring a white bulb color in onion (Allium Cepa L.) using bulked Segregant analysis and RNA-Seq. Mol Breeding 37:94. 10.1007/s11032-017-0697-6

[CR4] Cai XX, He WC, Qian Q, Shang LG (2025) Genetic resource utilization in wild rice species: genomes and gene bank. New Crops 2:100065. 10.1016/j.ncrops.2025.100065

[CR5] Che RH, Tong HN, Shi BH, Liu YQ, Fang SR, Liu DP, Xiao YH, Hu B, Liu LC, Wang HR, Zhao MF, Chu CC (2015) Control of grain size and rice yield by *GL2*-mediated brassinosteroid responses. Nat Plants 2:15195. 10.1038/nplants.2015.195 27250747 10.1038/nplants.2015.195

[CR6] Chen S, Zhou Y, Zhou Chen Y, Gu J (2018) Fastp: an ultra-fast all-in-one FASTQ preprocessor. Bioinformatics 34(17):i884–i890. 10.1093/bioinformatics/bty560 30423086 10.1093/bioinformatics/bty560 PMC6129281

[CR7] Cheng Q, Kong LH, Wang P, Huang T, Wu GL, Wang YN, Liu RQ, He XY, He HH, Bian JM (2021) Research progress of grain shape, grain weight and grain filling in rice (*Oryza sativa* L). Mol Plant Breed 3(4):1–13 (in Chinese with English abstract). 10.13271/j.mpb.021.001196

[CR8] Du YX, Ye C, Han PJ, Sheng YL, Li F, Sun HZ, Zhang J, Li J (2025) The molecular mechanism of transcription factor regulation of grain size in rice. Plant Sci 354:112434. 10.1016/j.plantsci.2025.112434 40023197 10.1016/j.plantsci.2025.112434

[CR9] Fan CC, Xing YZ, Mao HL, Lu TT, Han B, Xu CG, Li XH, Zhang QF (2006) *GS3*, a major QTL for grain length and weight and minor QTL for grain width and thickness in rice, encodes a putative transmembrane protein. Theoretical Appl Genet 112:1164–1171. 10.1007/s00122-006-0218-1 10.1007/s00122-006-0218-1 16453132

[CR10] Fujisaki S, Sugiyama A, Eguchi T, Watanabe Y, Hiraiwa H, Honma D, Saito T, Yasue H (2004) Analysis of a full-length cDNA library constructed from swine olfactory bulb for Elucidation of expressed genes and their transcription initiation sites. J Vet Med Sci 66:15–23. 10.1292/jvms.66.15 14960805 10.1292/jvms.66.15

[CR11] Gao XY, Zhang JQ, Zhang XJ, Zhou J, Jiang Z, Huang P, Tan ZB, Bao YM, Cheng J (2019) Rice *qGL3*/*OsPPKL1* functions with the *GSK3*/*SHAGGY*-like kinase *OsGSK3* to modulate brassinosteroid signaling. Plant Cell 31:1077–1093. 10.1105/tpc.18.00836 30923230 10.1105/tpc.18.00836 PMC6533024

[CR12] Guo JJ, Fan J, Hauser BA, Rhee SY (2016) Target enrichment improves mapping of complex traits by deep sequencing. G3-Genes genomes genetics. 6:67–77. 10.1534/g3.115.023671 10.1534/g3.115.023671 PMC4704726 26530422

[CR13] Han ZY, Li F, Qiao WH, Nong BX, Cheng YL, Zhang LF, Huang JF, Wang YY, Lou DJ, Ge JY, Xing M, Fan WY, Nie YM, Guo WL, Wang SZ, Liu ZR, Li DT, Zheng XM, Yang QW (2022) Identification of candidate genes and clarification of the maintenance of the green pericarp of weedy rice grains. Front Plant Sci 13:930062. 10.3389/fpls.2022.930062 35937328 10.3389/fpls.2022.930062 PMC9354532

[CR14] He L, Liang WH, Hu J, Zhao CF, Yao S, Chen T, Zhu Z, Zhao Q, Lu K, Zhao L (2023) Additive effects of QTLs/genes on rice grain size traits revealed by genetic comparisons. Rice Sci 30(3):171–175. 10.1016/j.rsci.2023.03.001

[CR16] Hu XB (2016) Breeding and cultivation techniques of new variety Shenliangyou 571 of hybrid rice. J Anhui Agricultural Sci 44(18):88–90 (in Chinese with English abstract). 10.3969/j.issn.0517-6611.2016.18.028

[CR15] Hu J, Wang YX, Fang YX, Zeng LJ, Xu J, Yu HP, Shi ZY, Pan J, Zhang D, Kang S (2015) A rare allele of *GS2* enhances grain size and grain yield in rice. Mol Plant 8:1455–1465. 10.1016/j.molp.2015.07.002 26187814 10.1016/j.molp.2015.07.002

[CR17] Hu ZJ, Lu SJ, Wang MJ, He HH, Sun L, Wang HR, Liu XH, Liang J, Sun JL, Xin X (2018) A novel QTL *qTGW3* encodes the GSK3/SHAGGY-like kinase OsGSK5/OsSK41 that interacts with OsARF4 to negatively regulate grain size and weight in rice. Mol Plant 11(5):736–749. 10.1016/j.molp.2018.03.005 29567449 10.1016/j.molp.2018.03.005

[CR19] Huang RY, Jiang LR, Zheng JS, Wang TS, Wang HC, Huang YM, Hong ZL (2013) Genetic bases of rice grain shape: so many genes, so little known. Trends Plant Sci 18(4):218–226. 10.1016/j.tplants.2012.11.001 23218902 10.1016/j.tplants.2012.11.001

[CR20] Huang XH, Yang SH, Gong JY, Zhao Y, Feng Q, Gong H, Li WJ, Zhan QL, Cheng BY, Xia JH, Chen N, Hao ZN, Liu KY, Zhu CR, Huang T, Zhao Q, Zhang L, Fan DL, Zhou CC, LU YQ, Weng QJ, Wang ZX, Li JY, Han B (2015) Genomic analysis of hybrid rice varieties reveals numerous superior alleles that contribute to heterosis. Nat Commun 6:6258–6267. 10.1038/ncomms7258 25651972 10.1038/ncomms7258 PMC4327311

[CR18] Huang K, Wang DK, Duan PG, Zhang BL, Xu R, Li N, Li YH (2017a) Wide and Thick *GRAIN* 1, which encodes an otubain-like protease with deubiquitination activity, influences grain size and shape in rice. Plant J 91:849–860. 10.1111/tpj.13613 28621888 10.1111/tpj.13613

[CR21] Huang Z, Peng G, Liu XJ, Deora A, Falk KC, Gossen BD, Mcdonald MR, Fu FQ (2017b) Fine mapping of a clubroot resistance gene in Chinese cabbage using SNP markers identified from bulked Segregant RNA sequencing. Front Plant Sci 8:1448. 10.3389/fpls.2017.01448 28894454 10.3389/fpls.2017.01448 PMC5581393

[CR22] Ishimaru K, Hirtsu N, Madoka Y, Murakami N, Hara N, Onodera H, Kashiwagi T, Ujiie K, Shimizu BI, Onishi A (2013) Loss of function of the IAA-glucose hydrolase gene *TGW6* enhances rice grain weight and increases yield. Nat Genet 45:707–711. 10.1038/ng.2612 23583977 10.1038/ng.2612

[CR23] Janzen GM, Wang L, Hufford MB (2019) The extent of adaptive wild introgression in crops. New Phytol 221:1279–1288. 10.1111/nph.15457 30368812 10.1111/nph.15457

[CR24] Jiang YF, Zhou MG, Ke SM, Deng XX, Li YS (2024) *GSW3.1*, a novel gene controlling grain size and weight in rice. Crop J 12(3):796–802. 10.1016/j.cj.2024.05.002

[CR25] Jin JH, Li SF, Zhao YD, Wang D, Zhang Y, Jin GG, Li HN, Quan C, Zhang Q (2025a) Study on important agronomic characters and genetic diversity of 1775 rice germplasm resources. J Plant Genetic Resour 26(3):481–495 (in Chinese with English abstract). 10.13430/j.cnki.jpgr.20240602002

[CR26] Jin YR, Chen B, Wang XK, Zhou TT, Li X, Deng JJ, Yang YW, Guo DS, Zhang BL (2025b) Generation of low-glutelin rice (*Oryza sativa* L.) germplasm through long fragment deletion using CRISPR/Cas9-mediated targeted mutagenesis. Scientia Agricultura Sinica 58(6):1052–1064. 10.3864/j.issn.0578-1752.2025.06.002

[CR27] Kang YW, Chen YY, Zhang YX (2020) Research progress and breeding prospects of grain size associated genes in rice. Chin J Rice Sci 34(6):3–14 (in Chinese with English abstract). 10.16819/j.1001-7216.2020.9135

[CR28] Kurlovs AH, Snoeck S, Kosterlitz O, Leeuwen TV, Clark RM (2019) Trait mapping in diverse arthropods by bulked Segregant analysis. Curr Opin Insect Sci 36:57–65. 10.1016/j.cois.2019.08.004 31499416 10.1016/j.cois.2019.08.004

[CR29] Langfelder P, Horvath S (2012) Fast R functions for robust correlations and hierarchical clustering. J Stat Softw 46:1–17. 10.18637/jss.v046.i11 23050260 PMC3465711

[CR30] Lei L, Zheng HL, Bi YL, Yang LM, Liu HL, Wang JG, Sun J, Zhao HW, Li XW, Li JM, Lai YC, Zou DT (2020) Identification of a major QTL and candidate gene analysis of salt tolerance at the bud burst stage in rice (Oryza sativa L.) using QTL-Seq and RNA-Seq. Rice 13:55–69. 10.1186/s12284-020-00416-1 32778977 10.1186/s12284-020-00416-1 PMC7417472

[CR38] Li ZQ, Xu YH (2021) Bulk segregation analysis in NGS era: a review for its teenage years. Plant J 11:1–20. 10.1111/tpj.15646 10.1111/tpj.15646 34931728

[CR32] Li H, Handsaker B, Wysoker A, Fennell T, Ruan J, Homer N, Marth G, Abecasis G, Durbin R (2009) The sequence alignment/map format and samtools. Bioinformatics 25(16):2078–2079. 10.1093/bioinformatics/btp352 19505943 10.1093/bioinformatics/btp352 PMC2723002

[CR37] Li YB, Fan CC, Xing YZ, Jiang YH, Luo LJ, Sun L, Shao D, Xu C, Li X, Xiao J (2011) Natural variation in *GS5* plays an important role in regulating grain size and yield in rice. Nat Genet 43:1266–1269. 10.1038/ng.977 22019783 10.1038/ng.977

[CR33] Li N, Xu R, Li YH (2019) Molecular networks of seed size control in plants. Annu Rev Plant Biol 70:435–463. 10.1146/annurev-arplant-050718-095851 30795704 10.1146/annurev-arplant-050718-095851

[CR35] Li PP, Zhu YJ, Guo L, Zhuang JY, Fan YY (2020) Fine mapping of *qGL1.1*, a minor QTL for grain length, using near isogenic lines derived from residual heterozygotes in rice. Chin J Rice Sci 34(2):125–134. 10.16819/j.1001-7216.2020.9125

[CR31] Li F, Han ZY, Qiao WH, Wang JR, Song Y, Cui YX, Li JQ, Ge HY, Lou DJ, Fan WY, Li DT, Nong BX, Zhang ZQ, Cheng YL, Zhang LF, Zheng XM, Yang QW (2021) High-quality genomes and high-density genetic map facilitate the identification of genes from a weedy rice. Front Plant Sci 12:775051. 10.3389/fpls.2021.775051 34868173 10.3389/fpls.2021.775051 PMC8639688

[CR34] Li P, Li G, Zhang YW, Zuo JF, Liu JY, Zhang YM (2022) A combinatorial strategy to identify various types of QTLs for quantitative traits using extreme phenotype individuals in an F_2_ population. Plant Commun 3(9):100319. 10.1016/j.xplc.2022.100319 35576159 10.1016/j.xplc.2022.100319 PMC9251438

[CR36] Li XX, Wu ME, Zhang JC, Xu JY, Diao YF, Li YB (2024) The *OsCLV2s-OsCRN1* co-receptor regulates grain shape in rice. J Genet Genomics 51(7):691–702. 10.1016/j.jgg.2024.03.011 38575110 10.1016/j.jgg.2024.03.011

[CR39] Liu J, Chen J, Zheng XM, Wu FQ, Lin QB, Heng YQ, Tian P, Cheng ZJ, Yu X, Zhou K (2017) *GW5* acts in the brassinosteroid signaling pathway to regulate grain width and weight in rice. Nat Plants 3:17043. 10.1038/nplants.2017.43 28394310 10.1038/nplants.2017.43

[CR40] Liu Q, Han RX, Wu K, Zhang JQ, YE YF, Wang SS, Chen JF, Pan YJ, Li Q, Xu XP, Zhou JW, Tao DY, Wu YJ, Fu XD (2018) G-protein βγ subunits determine grain size through interaction with MADS-domain transcription factors in rice. Nat Commun 9:852. 10.1038/s41467-018-03047-9 29487282 10.1038/s41467-018-03047-9 PMC5829230

[CR41] Lu XD, Li F, Xiao YH, Wang F, Zhang GL, Deng HB, Tang WB (2023) Grain shape genes: shaping the future of rice breeding. Rice Sci 30(5):379–404. 10.1016/j.rsci.2023.03.014

[CR42] Luo XB, Xu L, Wang Y, Dong JH, Chen YL, Tang MM, Fan LX, Zhu YL, Liu LW (2020) An ultra-high‐density genetic map provides insights into genome synteny, recombination landscape and taproot skin colour in radish (*Raphanus sativus* L). Plant Biotechnol J 18:274–286. 10.1111/pbi.13195 31218798 10.1111/pbi.13195 PMC6920339

[CR43] Luo YM, Chen YY, Xue P, Wang BF, Kang YW, Zhang Y, Chen DB, Hong YB, Wu WX, Liu QN, Zhan XD, Liu YJ, Cheng SH, Zhang YX, Cao LY (2025) Modulation of rice grain shape and appearance by the *GS10*-encoded long coiled-coil protein. Crop J 13(1):158–169. 10.1016/j.cj.2024.11.002

[CR44] Ma X, Fu YC, Zhao XH, Jiang LY, Zhu ZF, Gu P, Xu WY, Su Z, Sun C, Tan L (2016) Genomic structure analysis of a set of *Oryza* Nivara introgression lines and identification of yield-associated QTLs using whole-genome resequencing. Sci Rep 6:27425–27437. 10.1038/srep27425 27251022 10.1038/srep27425 PMC4890301

[CR45] Ma XD, Wang H, Yan S, Zhou GQ, Zhou KN, Zhang Q, Li MM, Yang Y, Li D, Song P (2025) Large-scale genomic and phenomic analyses of modern cultivars empower future rice breeding design. Mol Plant 18(4):651–668. 10.1016/j.molp.2025.03.007 40083159 10.1016/j.molp.2025.03.007

[CR46] Mao HL, Sun SY, Yao JL, Wang CR, Yu SB, Xu CG, Li XH, Zhang QF (2018) Linking differential domain functions of the *GS3* protein to natural variation of grain size in rice. Proceedings of the National Academy of Sciences 107: 19579–19584. 10.1073/pnas.1014419107 10.1073/pnas.1014419107 PMC2984220 20974950

[CR47] McKenna A, Hanna M, Banks E, Sivachenko A, Cibulskis K, Kernytsky A, Garimella K, Altshuler D, Gabriel S, Daly M (2010) The genome analysis toolkit: a mapreduce framework for analyzing next-generation DNA sequencing data. Genome Res 20:1297–1303. 10.1101/gr.107524.110 20644199 10.1101/gr.107524.110 PMC2928508

[CR48] Miao J, Wei XY, Cao CY, Sun JB, Xu YJ, Zhang Z, Wang QS, Pan YC, Wang Z (2024) Pig pangenome graph reveals functional features of non-reference sequences. J Anim Sci Biotechnol 15:32. 10.1186/s40104-023-00984-4 38389084 10.1186/s40104-023-00984-4 PMC10882747

[CR49] Ooijen JV, Ooijen WV, Van J, Ooijen J, Hoorn J, van Duin J JW (2009) MapQTL^®^6. Software for the mapping of quantitative trait loci in experimental populations of diploid species. Kyazma BV, Wageningen, The Netherlands

[CR50] Qi P, Lin YS, Song XJ, Shen JB, Huang W, Shan JX, Zhu MZ, Jiang LW, Gao JP, Lin HX (2012) The novel quantitative trait locus *GL3.1* control rice grain size and yield by regulating Cyclin-T1;3. Cell Res 22:1666–1680. 10.1038/cr.2012.151 23147796 10.1038/cr.2012.151 PMC3515756

[CR51] Shi HT, Lou C, Wang JF, Dong DQ, Yang LF, Li GZ, Tian ZQ, Han QX, Wang PF, Kang GZ (2024) Identification of P-efficient elite allele of the *TaPHT1;6* gene and development of its functional marker in common wheat (*Triticum aestivum* L). J Integr Agric 24(5):1646–1655. 10.1016/j.jia.2024.09.009

[CR52] Shomura A, Izawa T, Ebana K, Ebitani T, Kanegae H, Konishi S, Yano M (2008) Deletion in a gene associated with grain size increased yields during rice domestication. Nat Genet 40:1023–1028. 10.1038/ng.169 18604208 10.1038/ng.169

[CR53] Si LZ, Chen JY, Huang XH, Gong H, Luo JH, Hou QQ, Zhou TY, Lu TT, Zhu JJ, Shangguan YY, Chen EW, Gong CX, Zhao Q, Jing YF, Zhao Y, Li Y, Cui LL, Fan DL, Lu YQ, Weng QJ, Wang YC, Zhan QL, Liu KY, Wei XH, Kyungsook A, Gynheung A, Han B (2016) *OsSPL13* controls grain size in cultivated rice. Nat Genet 48:447–456. 10.1038/ng.3518 26950093 10.1038/ng.3518

[CR54] Song XJ, Huang W, Shi M, Zhu MZ, Lin HX (2007) A QTL for rice grain width and weight encodes a previously unknown RING-type E3 ubiquitin ligase. Nat Genet 39:623–630. 10.1038/ng2014 17417637 10.1038/ng2014

[CR55] Song XJ, Kuroha T, Ayano M, Furuta T, Nagai K, Segami S, Miura K, Ogawa D, Kamur T, Suzuki T, Higashiyama T, Yamasaki M, Mori H, Inukai Y, Wu JZ, Kitano H, Sakakibara H, Jacobsen SE, Ashikari M (2015) Rare allele of a previously unidentified histone H4 acetyltransferase enhances grain weight, yield, and plant biomass in rice. Proc Natl Acad Sci USA 112:76–81. 10.1073/pnas.1421127112 25535376 10.1073/pnas.1421127112 PMC4291654

[CR56] Song YY, Yang HZ, Zhu WR, Wang HL, Zhang JC, Li YB (2024) The Os14-3-3 family genes regulate grain size in rice. J Genet Genomics 51(4):454–457. 10.1016/j.jgg.2023.10.005 37913987 10.1016/j.jgg.2023.10.005

[CR57] Sun J, Ma DR, Tang L, Zhao MH, Zhang GC, Wang WJ, Song JY, Li X, Liu Z, Zhang W (2019) Population genomic analysis and de Novo assembly reveal the origin of weedy rice as an evolutionary game. Mol Plant 12(5):632–647. 10.1016/j.molp.2019.01.019 30710646 10.1016/j.molp.2019.01.019

[CR58] Takagi H, Abe A, Yoshida K, Kosugi S, Natsume S, Mitsuka C, Uemura A, Utsushi H, Tamiru M, Takuno S (2013) QTL-seq: rapid mapping of quantitative trait loci in rice by whole genome resequencing of DNA from two bulked populations. Plant J 74:174–183. 10.1111/tpj.12105 23289725 10.1111/tpj.12105

[CR59] Takai T, Oo AZ, Okamoto T, Nakano H (2025) MP3, a quantitative trait locus for increased panicle number, improves rice yield potential in Japan by connecting with high source and translocation traits. Field Crops Res 318:109566. 10.1016/j.fcr.2024.109566

[CR60] Tang YY, Huang Z, Xu SH, Zhou WJ, Ren JJ, Yu FX, Wang JS, Ma W, Qiao L (2024) Candidate genes conferring ethylene-response in cultivated peanuts determined by BSA-seq and fine-mapping. Crop J 12(3):856–865. 10.1016/j.cj.2024.03.003

[CR63] Wang HY, Yang FT, Liu L (2006) Comparison and application of standardized regressive coefficient and partial correlation coefficient. J Quant Technological Econ 23(9):150–155 (in Chinese with English abstract). 10.3969/j.issn.1000-3894.2006.09.016

[CR64] Wang SC, Basten C, Zeng Z (2012a) Windows QTL cartographer v2.5. Raleigh. North Carolina State University, NC

[CR66] Wang SK, Wu K, Yuan QB, Liu XY, Liu ZB, Lin XY, Zheng RZ, Zhu HT, Dong GJ, Qian Q, Zhang GQ, Fu XD (2012b) Control of grain size, shape and quality by *OsSPL16* in rice. Nat Genet 44:950–954. 10.1038/ng.2327 22729225 10.1038/ng.2327

[CR67] Wang YX, Xiong GS, Hu J, Jiang L, Yu H, Xu J, Fang YX, Zeng LJ, Xu EB, Xu J, Ye WJ, Meng XB, Liu RF, Chen HQ, Jing YH, Wang YH, Zhu XD, Li JY, Qian Q (2015a) Copy number variation at the *GL7* locus contributes to grain size diversity in rice. Nat Genet 47:944–948. 10.1038/ng.3346 26147619 10.1038/ng.3346

[CR65] Wang SK, Li S, Liu Q, Wu K, Zhang JQ, Wang SS, Wang Y, Chen XB, Zhang Y, Gao CX, Wang F, Huang HX, Fu XD (2015b) The *OsSPL16-GW7* regulatory module determines grain shape and simultaneously improves rice yield and grain quality. Nat Genet 47:949–954. 10.1038/ng.3352 26147620 10.1038/ng.3352

[CR61] Wang AH, Hou QQ, Si LZ, Huang XH, Luo JH, Lu DF, Zhu JJ, Shangguan YY, Miao J, Xie Y (2019) The PLATZ transcription factor *GL6* affects grain length and number in rice. Plant Physiol 180(4):2077–2090. 10.1104/pp.18.01574 31138620 10.1104/pp.18.01574 PMC6670106

[CR62] Wang FR, Zhang JX, Chen Y, Zhang CY, Gong JW, Song ZQ, Zhou J, Wang JJ, Zhao CJ, Jiao MJ, Liu AY, Du ZH, Yuan YL, Fan SJ, Zhang J (2020) Identification of candidate genes for key fibre-related QTLs and derivation of favourable alleles in Gossypium hirsutum Recombinant inbred lines with G.barbadense introgressions. Plant Biotechnol J 18:707–720. 10.1111/pbi.13237 31446669 10.1111/pbi.13237 PMC7004909

[CR68] Wen JQ, Jiang FL, Wen YQ, Sun MT, Shi XP, Zhou YZ, Yu L, Wu Z (2019) Identification of heat-tolerance QTLs and high-temperature stress-responsive genes through conventional QTL mapping, QTL-seq and RNA-seq in tomato. BMC Plant Biol 19:398–415. 10.1186/s12870-019-2008-3 31510927 10.1186/s12870-019-2008-3 PMC6739936

[CR70] Wu WG, Liu XY, Wang MH, Meyer RS, Luo XJ, Ndjiondjop MN, Tan LB, Zhang JW, Wu JZ, Cai HW, Sun CQ, Wang XK, Wang RA, Zhu ZF (2017) A single-nucleotide polymorphism causes smaller grain size and loss of seed shattering during African rice domestication. Nat Plants 3:17064. 10.1038/nplants.2017.64 28481332 10.1038/nplants.2017.64

[CR69] Wu DY, Qiu J, Sun J, Song BK, Olsen KM, Fan LJ (2022) Weedy rice, a hidden gold mine in the paddy field. Mol Plant 15(4):566–568. 10.1016/j.molp.2022.01.008 35032686 10.1016/j.molp.2022.01.008

[CR72] Xia D, Zhou H, Qiu L, Jiang HC, Zhang QL, Gao GJ, He YQ (2017) Mapping and verification of grain shape QTLs based on an advanced backcross population in rice. PLoS ONE 12(11):e0187553. 10.1371/journal.pone.0187553 29145412 10.1371/journal.pone.0187553 PMC5690652

[CR71] Xia D, Zhou H, Liu RJ, Dan WH, Li PB, Wu B, Chen JX, Wang LQ, Gao GJ, Zhang QL, He YQ (2018) *GL3.3*, a novel QTL encoding a *GSK3/SHAGGY*-like kinase, epistatically interacts with *GS3* to produce extra-long grains in rice. Mol Plant 11(5):754–756. 10.1016/j.molp.2018.03.006 29567448 10.1016/j.molp.2018.03.006

[CR73] Xie WB, Feng Q, Yu HH, Huang XH, Zhao Q, Xing YZ, Yu SB, Han B, Zhang Q (2010) Parent-independent genotyping for constructing an ultrahigh-density linkage map based on population sequencing. Proc Natl Acad Sci USA 107:10578–10583. 10.1073/pnas.1005931107 20498060 10.1073/pnas.1005931107 PMC2890813

[CR74] Xiong HY, Yu JP, Miao JL, Li JJ, Zhang HL, Wang X, Liu PL, Zhao Y, Jiang CH, Yin ZG, Li Y, Guo Y, Fu BY, Wang WS, Li ZK, Jauhar A, Li ZC (2018) Natural variation in *OsLG3* increases drought tolerance in rice by inducing ROS scavenging. Plant Physiol 178:451–467. 10.1104/pp.17.01492 30068540 10.1104/pp.17.01492 PMC6130013

[CR75] Xu MW, Xu C, Chen MZ, Xiao ZH, Wang YX, Xu Y, Xu DL (2023) Comparative analysis of commonly used bioinformatics software based on omics. Gene Rep 32:101800. 10.1016/j.genrep.2023.101800

[CR76] Yan J, Zou D, Li C, Zhang Z, Song SH, Wang XF (2020) SR4R: an integrative SNP resource for genomic breeding and population research in rice. Genom Proteom Bioinform 18(2):173–185. 10.1016/j.gpb.2020.03.002 10.1016/j.gpb.2020.03.002 PMC7646087 32619768

[CR80] Yang YS, Deng QY, Chen LY, Deng HB, Zhuang W, Xiong YD (2006) Yield-increasing effect of yield-enhancing QTL from Oryza rufipogon after being transferred into late-season rice restorer line. Mol Plant Breed 4(1):59–64 (in Chinese with English abstract). 10.3969/j.issn.1672-416X.2006.01.011

[CR77] Yang JH, Zhang CT, Zhao N, Zhang LL, Hu ZY, Chen S, Zhang MF (2018) Chinese root-type mustard provides phylogenomic insights into the evolution of the multi-use diversified allopolyploid brassica juncea. Mol Plant 11(3):512–514. 10.1016/j.molp.2017.11.007 29183772 10.1016/j.molp.2017.11.007

[CR79] Yang QW, Cheng YL, Zhang LF, Han ZY, Li F, Zhang WX, Qiao WH, Zheng XM (2022) Discovery and study of a green pericarp germplasm in rice. J Plant Genetic Resour 23(1):123–128 (in Chinese with English abstract). 10.13430/j.cnki.jpgr.20210610001

[CR78] Yang LM, Li P, Wang JG, Liu HL, Zheng HL, Xin W, Zou D (2023) Fine mapping and candidate gene analysis of rice grain length *QTL qGL9.1*. Int J Mol Sci 24(14):11447. 10.3390/ijms241411447 37511217 10.3390/ijms241411447 PMC10380290

[CR81] Ying JZ, Ma M, Bai C, Huang XH, Liu JL, Fan YY, Song XJ (2018) *TGW3*, a major QTL that negatively modulates grain length and weight in rice. Mol Plant 11(5):750–753. 10.1016/j.molp.2018.03.007 29567450 10.1016/j.molp.2018.03.007

[CR82] Yu JP, Xiong HY, Zhu XY, Zhang HL, Liu HH, Miao JL, Wang WS, Tang ZS, Zhang ZY, Yao GX, Zhang Q, Pan YH, Wang X, Rashid MAR, Li JJ, Gao YM, Li ZK, Yang WC, Fu XD, Li ZC (2017) *OsLG3* contributing to rice grain length and yield was mined by Ho-LAMap. BMC Biol 15:1–28. 10.1186/s12915-017-0365-7 28385155 10.1186/s12915-017-0365-7 PMC5383996

[CR83] Yu ZC, Chen YM, Zhou Y, Zhang YL, Li MY, Ouyang YR, Chebotarov D, Mauleon R, Zhao H, Xie WB (2023) Rice gene index: A comprehensive pan-genome database for comparative and functional genomics of Asian rice. Mol Plant 16(5):798–801. 10.1016/j.molp.2023.03.012 36966359 10.1016/j.molp.2023.03.012

[CR84] Zhan PL, Ma SP, Xiao ZL, Li FP, Wei X, Lin SJ, Wang XL, Ji Z, Fu Y, Pan JH, Zhou M, Liu Y, Chang ZY, Li L, Bu SH, Liu ZP, Zhu HT, Liu GF, Zhang GQ, Wang SK (2022) Natural variations in grain length 10 (GL10) regulate rice grain size. J Genet Genomics 49(5):405–413. 10.1016/j.jgg.2022.01.008 35151907 10.1016/j.jgg.2022.01.008

[CR85] Zhang LY, Li HH, Wang JK (2015a) Linkage analysis and map construction in genetic populations of clonal F1 and double cross. G3-Genes genomes genetics. 5:427–439. 10.1534/g3.114.016022 10.1534/g3.114.016022 PMC4349096 25591919

[CR86] Zhang YD, Zheng J, Liang ZK, Liang YL, Peng ZH, Wang CL (2015b) Verification and evaluation of grain QTLs using RILs from TD70 x Kasalath in rice. Genet Mol Res 14(4):14882–14892. 10.4238/2015.November.18.53 26600549 10.4238/2015.November.18.53

[CR88] Zhao MF, Li XH, Yang JB, Xu CG, Hu RY, Liu DJ, Zhang Q (1999) Relationship between molecular marker heterozygosity and hybrid performance in intra- and inter-subspecific crosses of rice. Plant Breeding 118:139–144. 10.1046/j.1439-0523.1999.118002139.x

[CR87] Zhao DS, Li QF, Zhang CQ, Zhang C, Yang QQ, Pan LX, Ren XY, Lu J, Gu MH, Liu QQ (2018) GS9 acts as a transcriptional activator to regulate rice grain shape and appearance quality. Nat Commun 9:1240. 10.1038/s41467-018-03616-y 29588443 10.1038/s41467-018-03616-y PMC5869696

