# Supplementary material for: Fine Mapping and Candidate Gene Identification of ORUFILM03g000096 Gene in Weedy Rice LM8: Insights into Grain Length Regulation
Source: Rice (N Y). 2025 Nov 5;18:101. doi: 10.1186/s12284-025-00858-5 (PMC12589740; doi:10.1186/s12284-025-00858-5)
Supplement: Supplementary file 1 — Additional file 1. [file 12284_2025_858_MOESM1_ESM.pdf]

Table S1 Location and annotation of the seven genes in the candidate QTL interval.

Chromosome	Gene	RAP_Locus	Gene annotation
LG03	*ORUFILM03g000090*	-	-
LG03	*ORUFILM03g000091*	*Os03g0428200*	terpene synthase, N-terminal domain containing protein, expressed
LG03	*ORUFILM03g000092*	-	-
LG03	*ORUFILM03g000093*	-	-
lg03	*ORUFILM03g000094*	-	-
LG03	*ORUFILM03g000095*	*Os03g0427900*	U-box domain-containing protein, putative, expressed
LG03	*ORUFILM03g000096*	*Os03g0427300*	glutelin, putative, expressed

Table S2 Variation in InDels within the candidate QTL interval.

Genetic elements	Location/nearest Gene	Chromosome	Starting position	Termination position	REF	ALT
upstream	*ORUFILM03g000096*	LG03	18807884	18807893	TAGTTTGTTT	-
upstream	*ORUFILM03g000096*	LG03	18808008	18808008	-	T
upstream	*ORUFILM03g000096*	LG03	18808239	18808240	AT	-
intronic	*ORUFILM03g000096*	LG03	18809415	18809415	-	TA
intergenic	*ORUFILM03g000096_ORUFILM03g000095*	LG03	18811641	18811642	AT	-
intergenic	*ORUFILM03g000096_ORUFILM03g000095*	LG03	18811759	18811759	T	-
intergenic	*ORUFILM03g000096_ORUFILM03g000095*	LG03	18812377	18812379	TAT	-
intergenic	*ORUFILM03g000096_ORUFILM03g000095*	LG03	18812626	18812626	-	GAT
intergenic	*ORUFILM03g000096_ORUFILM03g000095*	LG03	18812698	18812698	A	-
intergenic	*ORUFILM03g000096_ORUFILM03g000095*	LG03	18812718	18812718	A	-
intergenic	*ORUFILM03g000096_ORUFILM03g000095*	LG03	18812826	18812827	TT	-
intergenic	*ORUFILM03g000096_ORUFILM03g000095*	LG03	18813249	18813249	-	T
intergenic	*ORUFILM03g000096_ORUFILM03g000095*	LG03	18813987	18813987	-	GAG
intergenic	*ORUFILM03g000096_ORUFILM03g000095*	LG03	18814229	18814229	T	-
intergenic	*ORUFILM03g000096_ORUFILM03g000095*	LG03	18815585	18815585	-	GCCTTAACT
intergenic	*ORUFILM03g000096_ORUFILM03g000095*	LG03	18821458	18821458	-	A
intergenic	*ORUFILM03g000096_ORUFILM03g000095*	LG03	18821556	18821556	-	T
intergenic	*ORUFILM03g000096_ORUFILM03g000095*	LG03	18825914	18825914	-	GA
intronic	*ORUFILM03g000095*	LG03	18830829	18830829	A	-
intronic	*ORUFILM03g000095*	LG03	18831744	18831744	-	T
intergenic	*ORUFILM03g000095_ORUFILM03g000094*	LG03	18835315	18835315	-	C
downstream	*ORUFILM03g000094*	LG03	18838361	18838362	AT	-
upstream	*ORUFILM03g000092*	LG03	18843145	18843145	T	-

Genetic elements	Location/nearest Gene	Chromosome	Starting position	Termination position	REF	ALT
intronic	*ORUFILM03g000091*	LG03	18848989	18848989	-	AAG
intronic	*ORUFILM03g000091*	LG03	18849186	18849186	-	GGA
intronic	*ORUFILM03g000091*	LG03	18849211	18849211	-	AGTAGTA
intronic	*ORUFILM03g000091*	LG03	18849419	18849419	-	A
intronic	*ORUFILM03g000091*	LG03	18849536	18849536	G	-
intronic	*ORUFILM03g000091*	LG03	18853032	18853032	G	-
intronic	*ORUFILM03g000091*	LG03	18854387	18854387	-	CTCTCTCT
intronic	*ORUFILM03g000091*	LG03	18854410	18854411	TG	-
intronic	*ORUFILM03g000091*	LG03	18854920	18854920	-	A
intronic	*ORUFILM03g000091*	LG03	18855038	18855038	-	G
intergenic	*ORUFILM03g000091_ORUFILM03g000090*	LG03	18857886	18857886	-	TA
intergenic	*ORUFILM03g000091_ORUFILM03g000090*	LG03	18858094	18858094	-	C
intergenic	*ORUFILM03g000091_ORUFILM03g000090*	LG03	18858706	18858706	-	AA
intergenic	*ORUFILM03g000091_ORUFILM03g000090*	LG03	18860255	18860263	CTGGTTTAG	-
intergenic	*ORUFILM03g000091_ORUFILM03g000090*	LG03	18860865	18860865	-	A
intergenic	*ORUFILM03g000091_ORUFILM03g000090*	LG03	18861061	18861070	GCCCAGCCCG	-
intergenic	*ORUFILM03g000091_ORUFILM03g000090*	LG03	18864077	18864083	CAAATGT	-
intronic	*ORUFILM03g000090*	LG03	18868430	18868430	C	-
intronic	*ORUFILM03g000090*	LG03	18869988	18869988	T	-
intronic	*ORUFILM03g000090*	LG03	18870014	18870014	-	GATT
intronic	*ORUFILM03g000090*	LG03	18870460	18870460	-	TAAACTAAA
intronic	*ORUFILM03g000090*	LG03	18870481	18870481	-	A
intronic	*ORUFILM03g000090*	LG03	18870682	18870682	-	T
intronic	*ORUFILM03g000090*	LG03	18870872	18870886	CTAAAGTTTATAGAC	-
intronic	*ORUFILM03g000090*	LG03	18872563	18872563	-	AG
intronic	*ORUFILM03g000090*	LG03	18873493	18873493	A	-
intronic	*ORUFILM03g000090*	LG03	18873535	18873535	-	T
intronic	*ORUFILM03g000090*	LG03	18873855	18873855	A	-
